# Supplementary material for: Similarity of TOPSIS results based on criterion variability: Case study on public economic
Source: PLoS One. 2022 Aug 4;17(8):e0271951. doi: 10.1371/journal.pone.0271951 (PMC9352064; doi:10.1371/journal.pone.0271951)
Supplement: Attachment — Submitted filename: Response to Reviewers.pdf [file pone.0271951.s002.pdf]

Journal: PLOS One
Manuscript ID: PONE-D-21-35126
Manuscript title: Similarity of TOPSIS Results based on Criterion Variability: Case Study on Public Economic

Reviewer #1

C1: This manuscript has high innovative value in method, but the language expression is more obscure. It is suggested that the authors should revise the language and grammar problems, and this manuscript should be reviewed again.
R: Thank you very much. The manuscript has undergone extensive and professional language proofreading, which has been carried out by an external entity.

Reviewer #2

C1: The authors are recommended to construct a framework of MCDM methods and show where TOPSIS is situated in this framework. Instead of categorising the methods based on where they were originated (c.f. the 2nd paragraph of Introduction), the authors are recommended to classify them according to the methodology (e.g.parametric/non-parametric; pairwise/threshold, etc.; c.f. Diaz-Balteiro et al., 2017).
R: The framework of MCDM methods is created and added in the Introduction. The recommended source is also added in the references.

C2: The authors mention a considerable number of MCDM methods in Introduction but explain little about the applicability and feasibility of each method. A recommendation is that authors answer the following questions to justify the selection of TOPSIS: Which scenario(s) is each method to be used for? What is the context and features of the problem you are to solve in this paper (i.e. the sample case)?
R: The applicability and feasibility of each method discussed in the Introduction is described via 2 new classifications, this way the selection of TOPSIS is explained. The sample case is described too.

C3: In Equation (4), why PIS and NIS are function of weight (w) rather than weighted normalised value (v)?
R: Thanks for reporting an error in the formula. The error is fixed.

C4: As is shown in Equation (5), Euclidean distance is computed and thereby the correlation between criteria is uncaptured. The authors are recommended to discuss on this.
R: Euclidean distance is the square root of the sum of squared differences between corresponding elements of the two vectors. It's a reverse measure of similarity: the smaller the Euclidean distance, the greater the similarity between X and Y. Euclidean distance is appropriate for data measured on the same scale that is our case (see criteria R1-R6). Correlation coefficient is (inversely) related to the Euclidean distance when the variables have been standardized, removing differences in scale.

C5: Please define the problem (L.195 – L.293) in a concise manner and focus the pointers relevant to the applicability and feasibility of TOPSIS.
R: The problem is defined in the context of TOPSIS and its applicability and feasibility.

C6: The authors are recommended to state the contribution of this paper in an explicit manner. The findings of this paper are explicit (c.f. Conclusion), but what are the contributions of these findings? Are they relevant to the long-standing criticism of TOPSIS like rank reversal (Shin et al., 2013)?
R: The contribution of this manuscript is added/defined in an explicit manner.

C7: Please show the author's name and year of publication in the in-text citation to improve the readability.
R: We totally agree with you that the author's name and year of publication in the in-text citation improve the readability. The manuscript is prepared according to the formal requirements of the journal, which requires the authors to be listed using numbers.

C8: Please add captions to the graphics.
R: Captions are added to each figure.

C9: Proofreading is highly recommended.
R: The manuscript has undergone extensive and professional language proofreading, which has been carried out by an external entity.
