# Supplementary material for: Similarity of TOPSIS results based on criterion variability: Case study on public economic
Source: PLoS One. 2022 Aug 4;17(8):e0271951. doi: 10.1371/journal.pone.0271951 (PMC9352064; doi:10.1371/journal.pone.0271951)
Supplement: Attachment — Submitted filename: PONE-D-21-35126.docx [file pone.0271951.s001.docx]

Similarity of TOPSIS Results based on Criterion Variability: Case Study on Public Economic

Focused on the MCDM method, this paper quantifies the differences in assessment with aid of TOPSIS and determines the priority of criteria with absolute and relative variability. No evidence shows that the method determining the variability of criteria has direct impact on the order of alternatives. Further, the low Jaccard index indicates low concurrence in the order of application of the TOPSIS technique in combination with both approaches. Kindly find the specific comments as below.

The authors are recommended to construct a framework of MCDM methods and show where TOPSIS is situated in this framework. Instead of categorising the methods based on where they were originated (c.f. the 2^nd^ paragraph of Introduction), the authors are recommended to classify them according to the methodology (e.g. parametric/non-parametric; pairwise/threshold, etc.; c.f. Diaz-Balteiro et al., 2017).

Diaz-Balteiro, L., Gonzalez-Pachon, J., & Romero, C. (2017). Measuring systems sustainability with multi-criteria methods: A critical review. *European Journal of Operational Research, 258*(2), 607-616. doi:10.1016/j.ejor.2016.08.075

The authors mention a considerable number of MCDM methods in Introduction but explain little about the applicability and feasibility of each method. A recommendation is that authors answer the following questions to justify the selection of TOPSIS: Which scenario(s) is each method to be used for? What is the context and features of the problem you are to solve in this paper (i.e. the sample case)?

In Equation (4), why PIS and NIS are function of weight (w) rather than weighted normalised value (v)?

As is shown in Equation (5), Euclidean distance is computed and thereby the correlation between criteria is uncaptured. The authors are recommended to discuss on this.

Please define the problem (L.195 – L.293) in a concise manner and focus the pointers relevant to the applicability and feasibility of TOPSIS.

The authors are recommended to state the contribution of this paper in an explicit manner. The findings of this paper are explicit (c.f. Conclusion), but what are the contributions of these findings? Are they relevant to the long-standing criticism of TOPSIS like rank reversal (Shin et al., 2013)?

Please show the author’s name and year of publication in the in-text citation to improve the readability.

Please add captions to the graphics.

Proofreading is highly recommended.
