# Supplementary material for: Similarity of TOPSIS results based on criterion variability: Case study on public economic
Source: PLoS One. 2022 Aug 4;17(8):e0271951. doi: 10.1371/journal.pone.0271951 (PMC9352064; doi:10.1371/journal.pone.0271951)
Supplement: Attachment — Submitted filename: Response to Reviewers.docx [file pone.0271951.s003.docx]

Journal:	PLOS One

Manuscript ID:	PONE-D-21-35126R1

Manuscript title:	Similarity of TOPSIS Results based on Criterion Variability: Case Study on Public Economic

**Reviewer #1**

**C1**: The authors have done a good job of revision, and the paper meets the publication requirements of this journal.

**R**: Thank you very much for appreciating the adjustments made and the time devoted to it.

**Reviewer #2**

**C1**: The authors made significant revisions, which improves the readability of the manuscript. However, the manuscript needs better organisation. The authors put the detailed taxonomy of MCDM methods in the Introduction, which makes the Introduction less focused. For instead, the authors are recommended to elaborate on the taxonomy (some charts will improve the readability) in a following section and to justify the feasibility of TOPSIS thereby. You might go through the taxonomy (only one metric for classification but do not put origin and methods together) in Introduction in a succinct way but focus on the main issue and contribution of this manuscript in the Introduction.

**R**: Thank you very much for appreciating the adjustments made. The Introduction is rewritten. Origin description is deleted, only one metric for classification is described using a chart. With these changes (also text reducing), more emphasis is placed on the feasibility of TOPSIS and we more focus on the main issue and contribution of our manuscript.
